# High-Definition Transcranial Direct Current Stimulation in the Right Ventrolateral Prefrontal Cortex Lengthens Sustained Attention in Virtual Reality

**DOI:** 10.3390/bioengineering10060721

**Published:** 2023-06-14

**Authors:** Shan Yang, Ganbold Enkhzaya, Bao-Hua Zhu, Jian Chen, Zhi-Ji Wang, Eun-Seong Kim, Nam-Young Kim

**Affiliations:** 1 RFIC Center, Department of Electronic Engineering, Kwangwoon University, Nonwon-gu, Seoul 01897, Republic of Koreacnjacobiii@hotmail.com (J.C.); 2 NDAC Center, Department of Electronic Engineering, Kwangwoon University, Nonwon-gu, Seoul 01897, Republic of Korea; 3 Department of Pediatrics, Severance Children’s Hospital, Yonsei University, Seoul 03722, Republic of Korea

**Keywords:** event-related potential, hierarchical drift-diffusion model, right ventrolateral prefrontal cortex, sustained attention, transcranial direct current stimulation, virtual reality

## Abstract

Due to the current limitations of three-dimensional (3D) simulation graphics technology, mind wandering commonly occurs in virtual reality tasks, which has impeded it being applied more extensively. The right ventrolateral prefrontal cortex (rVLPFC) plays a vital role in executing continuous two-dimensional (2D) mental paradigms, and transcranial direct current stimulation (tDCS) over this cortical region has been shown to successfully modulate sustained 2D attention. Accordingly, we further explored the effects of electrical activation of the rVLPFC on 3D attentional tasks using anodal high-definition (HD)-tDCS. A 3D Go/No-go (GNG) task was developed to compare the after effects of real and sham brain stimulation. Specifically, GNG tasks were periodically interrupted to assess the subjective perception of attentional level, behavioral reactions were tracked and decomposed into an underlying decision cognition process, and electroencephalography data were recorded to calculate event-related potentials (ERPs) in rVLPFC. The *p*-values statistically indicated that HD-tDCS improved the subjective mentality, led to more cautious decisions, and enhanced neuronal discharging in rVLPFC. Additionally, the neurophysiological P300 ERP component and stimulation being active or sham could effectively predict several objective outcomes. These findings indicate that the comprehensive approach including brain stimulation, 3D mental paradigm, and cross-examined performance could significantly lengthen and robustly compare sustained 3D attention.

## 1. Introduction

As a simulation expertise that goes beyond conventional flat-panel displays, three-dimensional (3D) virtual reality (VR) adopts head tracking and binocular stereovision to synchronously change and render visual graphics, stereoscopically presenting a 360-degree artificial world from the egocentric perspective [1]. This advanced display technology for immersive man–machine interaction has gained application in the fields of education, healthcare and medical treatment, engineering and robotics, and occupational security [2]. In this context, sustained attention—the ability to orient cognition toward the external environment and amplify relevant objective cues for better behavioral decisions over extended periods—plays a crucial role in training and learning efficiency in the VR ecosystem [3].

It is well-known that it can be difficult to uninterruptedly concentrate one’s stream of thought on the current consideration, working memory, and further action decisions, as they always stray into the task-unrelated inner world, i.e., mind wandering [4]. Moreover, visually induced motion sickness (VIMS), characterized by symptoms of fatigue, headache, and nausea, often manifests after a certain duration of VR time, exacerbating the distractions and resulting in sluggish or impulsive reactions in the VR setting [5]. Sensory conflict has been proposed as the likely cause of VIMS, as the motion-simulating visual stimuli in VR induce a mismatch between the neural inputs generated by the vestibular system and the stored motor neural pattern [6]. Currently, one of the most popular approaches to alleviating virtual vision-elicited adverse symptoms involves limiting the field of vision; however, this comes at the cost of reduced visual perception and immersion, which can also degrade individual behaviors and responses [6,7,8]. Accordingly, facilitating sustained attention without affecting user experience and performance in the virtual environment (VE) remains a challenge, especially in the context of boosting the applications of VR in clinical or tutorial settings.

Apart from advancements in VR technology, brain stimulation could be another approach to dampening mind wandering in the VE. Because of the minimal occurrence of adverse effects and its ease of use, transcranial direct current stimulation (tDCS) which can noninvasively modulate the excitability of target neurons with an after effect has been used to mediate neuroplasticity and improve cognition [9,10]. The functions of the prefrontal cortex include execution, advanced cognition, and decision making through top-down control over other cerebral regions; it is therefore of great interest as a stimulation region [11]. In continuous performance tasks, the right ventrolateral prefrontal cortex (rVLPFC) is one of the notably active cortical regions at the onset of behavior-relevant stimuli for capturing exogenous cues, updating motor action, and inhibiting the shift of attention [12,13,14]. Previous research has indicated that tDCS over the rVLPFC can positively impact the attentional capacity of participants in the two-dimensional (2D) psychological paradigms displayed on paper or computer screens [15,16,17]. This suggests that tDCS neuromodulation strongly activates the rVLPFC to win the cerebral signal competition between willful focus and spontaneous distractions in a top-down manner, with more neurophysiological resources devoted to recruiting attentional networks and executing the task [18]. However, the issue of whether electrical stimulation over rVLPFC can likewise prolong sustained attention in 3D VE has not been addressed. Methodologies to represent and compare the level of sustained 3D attention need to be developed as well.

In this study, we adopted anodal high-definition (HD) tDCS to focally stimulate the rVLPFC of 10 healthy participants. Subsequently, subjects performed the developed 3D Go/No-go (GNG) tasks in VR, during which we cross-examined the subjective reports of sustained attention, behavioral outcomes including accuracy and reaction time (RT), and physiological electroencephalography (EEG) recordings. We statistically compared multiple results under the sham and stimulation conditions to explore the offline stimulation effects. Moreover, we derived regression models to assess the difference between the effects of active and sham stimulation and also the influence of the observed ERPs on subjective and objective measures of task performance. Finally, we ascertained that anodal tDCS over the rVLPFC could activate this area and thus extend the duration of sustained attention in a 3D VE. To the best of our knowledge, this is the first study to explore the positive effect of tDCS over rVLPFC on the 3D attentional capacity and further characterize the sustained attention in the VE.

## 2. Materials and Methods

### 2.1. Participants

The study protocol was designed in accordance with the Declaration of Helsinki and approved by the Institutional Review Board (IRB) of Kwangwoon University, Seoul, Republic of Korea (IRB No. 7001546-202300614-HR(SB)-005-01).

Ten (*n* = 10) healthy and right-handed male adults (mean age, 24.3 years; standard deviation, 1.5 years) were enrolled for participation in this study. The sex of participants was controlled because sex-related anatomical differences can significantly impact the tDCS-induced electrical field and perturb stimulation outcomes [19]. Prior to the actual experiment, we explained the research objectives and procedures to each participant, and then obtained signed informed consent forms from them. A large effect size could be estimated based on Cohen’s d values in paired-sample t-tests for all ERP comparisons (>0.8) and the adjusted R^2^ values in all regression models (>0.5) [20].

### 2.2. Procedure and Stimulation

**Figure 1:**
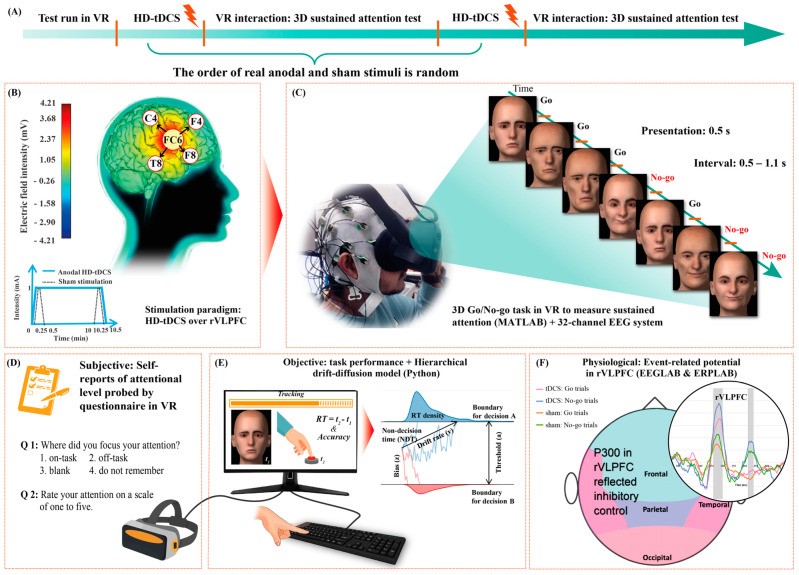
Schematic depiction of the experimental protocol. (**A**) Sequence of experimental phases; (**B**) real and sham high-definition transcranial direct current stimulation (HD-tDCS) interventions; (**C**) integration of the 32-channel electroencephalography (EEG) system and virtual reality (VR) setup to measure three-dimensional (3D) sustained attention after stimulation; (**D**) subjective reports of attentional level were obtained using a questionnaire displayed in VR after every 10 Go/No-go (GNG) task trials; (**E**) objective measurements of sustained attention were obtained by tracking performance in the GNG tasks in terms of reaction time (RT) and accuracy using a VR- and keyboard-connected computer, and further decomposing behavioral metrics into the hierarchical drift-diffusion model; and (**F**) sustained attention was physiologically analyzed based on the averaged ERPs of the FC6, F4, F8, C4, and T8 channels in the right ventrolateral prefrontal cortex (rVLPFC) using EEG data.

As illustrated in Figure 1A, initially, participants performed a pilot run of the 3D GNG task to train for about 5 min. Thereafter, they received real and sham stimulation, whose order was counterbalanced and randomized across participants [21,22]. In each condition, 4 × 1 HD-tDCS was applied using the battery-driven 8-channel Starstim^®^ neurostimulator system (Neuroelectrics, Barcelona, Spain) to achieve highly focal stimulation [23]. The anode was centrally positioned at FC6 with four return electrodes in a ring configuration at F4, F8, C4, and T8 [24]. In the real tDCS condition, the 1 mA electrical current was delivered for 10.5 min (fade-in and -out, 15 s). Conversely, in the sham condition, the 1 mA current ramped up and immediately down during both the first and last 30 s of stimulation (Figure 1B). Thus, single blinding was achieved, as participants experienced a tingling sensation at the two ends of the stimulation periods in both conditions [25]. Previous research has shown that a current intensity of 1 mA and stimuli duration of 9 to 13 min can elicit an after effect for 1 to 1.5 h [26,27]. Here, the GNG tasks were conducted 20 min after the stimulus ended and lasted for up to about 30 min; therefore, the stimulation after-effect period covered the participant performance duration (Figure 1C). During VR tasks, subjective reports of sustained attention in VR were obtained using a questionnaire (Figure 1D); objective results of response accuracy and RT were automatically recorded by the computer (Figure 1E); and physiological EEG data were measured to calculate the event-related potentials (ERPs) in the rVLPFC (Figure 1F). Participants rested for 2 h between the two conditions to exclude disturbances due to the prior stimulation.

### 2.3. VR and 3D GNG Task

The Oculus Quest VR headset (The Meta Inc., Menlo Park, CA, USA) was used to display the GNG program, and a wireless keyboard was used to obtain the participant responses. The 3D GNG tasks were developed via the Psychtoolbox (version 3.0.18) in MATLAB (version R2021a, The Mathworks Inc., Natick, MA, USA) and consisted of training and formal tests. We extracted eight 3D face models (i.e., two female happy, female sad, male happy, and male sad faces each) as trials from the database provided by Hays et al. [28]. Three-dimensional vision was achieved by rendering virtual views of the left and right 60-degree perspectives in the Oculus stereo glasses. The environment for face immersion was set as pure black. A random number algorithm was used to randomize the order of trials each time the program ran, during which the trial information and button responses were synchronously recorded by the Psychtoolbox. At the beginning of the tests, an instruction frame in VR reminded the participants to press the spacebar as quickly as possible for sad faces (Go trials) but to avoid any reaction to the happy faces (No-go trials). A brief training session preceded the formal experiment to familiarize participants with the procedure, avoiding the interference of non-proficiency in the first task (detailed information provided in the Supplementary Material). The ratio of No-go to Go trials in the formal test (400 trials) was 3:7. Each trial was presented for 0.5 s if no response was detected, whereas the face would disappear immediately once participants pressed the spacebar. The interval between trials was 0.5–1.1 s (random uniform jitter) to avoid rigid rhythm reduced attention.

After every 10 trials, a questionnaire with two questions interrupted the test to probe perceived attention in that short period. The first question was “Where did you focus your attention?” and participants could choose one of the following options: (1) on the task, (2) off the task, (3) blank, and (4) do not remember. The second question asked participants to rate their attention on a 5-point scale (from 1 = “not focused on the task at all” to 5 = “fully focused on the task”) to further index subjective attention.

### 2.4. Behavioral Analyses 

Go trials were marked as misses if participants did not press the spacebar within 1 s of trial onset. Conversely, a false reaction was marked for any No-go trial in which the spacebar was pressed within this period. RT was defined as the difference between the timestamps of button press and stimulus onset.

The hierarchical drift-diffusion model (HDDM), which is based on decision field theory, can be applied beyond superficial analyses of behavioral indicators to evaluate underlying dynamic cognition of decision making in an attentional task [29]. This algorithm applies sequential sampling to decompose RT distributions and reaction outcomes into robust parameters for assessing sensory information processing [30]. We utilized the HDDM (version 0.8.0) package in Python (version 3.8.5) to model the full posterior distribution of HDDM parameters. First, we chose the stimulus-coding function to estimate the response deviation between Go and No-go trials. Subsequently, we employed the Markov chain Monte Carlo (MCMC) approach as the sampling strategy and discarded the initial 500 samples as burn-in to eliminate model autocorrelation. We then applied Geweke’s statistic and plotted the trace, autocorrelation, and marginal posterior of each parameter to assess the convergence of the MCMC chain [31]. After confirming proper model convergence, we finally quantified the speed to extract information from a No-go and Go trial (drift rates, [v_nogo_] and [v_go_]), the time for pre-stimulus encoding and motor planning (non-decision time, [NDT]), the amount of evidence to decide (decision threshold, [a]), and the subjective initial bias of whether to press the spacebar (decision bias, [z]) [32].

### 2.5. EEG and ERP

Brain wave signals were recorded and amplified using a 32-channel actiCAP EEG system (Brain Products, Munich, Germany) [33]. The ground and online reference electrodes were positioned at the FPz and Fz, respectively. Two prefrontal electrodes (at FP1 and FP2) were excluded from the analysis as they were being physically pressed by the VR eyeshade and were providing poor quality data. The remaining 29 EEG electrodes were sampled at 500 Hz.

We analyzed the EEG data in MATLAB in combination with the EEGLAB (v2021.0) and ERPLAB (v8.30) packages [34,35]. EEG data were re-referenced to the Pz, which is geographically further away from the rVLPFC than Fz [36,37,38]. The IIR Butterworth and Parks–McClellan notch filters were used to band-pass data between 2–80 Hz and remove 60 Hz circuit noise, respectively [39,40]. Epochs were extracted from −200 ms before to 800 ms after 3D face display onsets. To block the interference of artifacts caused by eye movement, heartbeats, head movement, etc., we first performed independent component analysis, referred to the ICLabel algorithm to delete artifact components, and then visually inspected the scroll EEG data to manually remove epochs containing residual artifacts and noises [39,41,42]. All reserved epochs were baseline corrected.

ERPs can be used to quantify stimulation-triggered regional neuronal excitability with good temporal resolution [43,44]. According to the existing literature on ERP, the stimulation-locked P300 component associated with the rVLPFC triggers the outright inhibition in response to stop signals [17,45,46,47]. Hence, the P300 here was used to evaluate the level of inhibition reaction and mirror the tDCS-evoked functional activation of the rVLPFC. As described in detail in Supplementary Figure S1, the collapsed localizers approach was used to unbiasedly define the time windows for the ERP components, and ERPs were robustly quantified by the mean amplitude in these windows [48,49]. P300 amplitudes were averaged in the 270–308 ms time range and across the FC6, F4, F8, C4, and T8 channels to compare them between the two stimulation conditions for the Go and No-go trials. Additionally, another positive deflection at P400 was observed in the No-go trials, and its amplitudes were averaged in the 426–448 ms time range and across rVLPFC channels. Finally, we also depicted the mean scalp topographies in the aforementioned time windows to represent the corresponding energy distributions and confirm that the ERP analyses in pooled rVLPFC electrodes were plausible.

### 2.6. Statistical Analyses

The Wilcoxon signed-rank tests were used to determine whether the within-subject factor “stimulation type” affected the percentage of “on the task” options in mental feedback, the averaged mental scores, missed Go trials, false reactions in No-go trials, averaged RT for correct Go trials, and averaged RT for incorrect No-go trials. Regarding the contrasts in HDDM parameters, Bayesian estimation in Python can directly provide the statistically meaningful probabilities of the relationships. The paired-sample t-tests were used to compare the difference in ERP amplitudes between paired stimulation conditions; the effect sizes were reported in terms of Cohen’s d values, and the Shapiro–Wilk normalization tests were used to validate the assumption that the ERP difference between the two conditions followed a Gaussian distribution, i.e., *p* > 0.05 could prove normalized distribution. Differences in scalp topography were detected based on permutation-based statistics in EEGLAB. Additionally, hierarchical multiple regression was adopted to first estimate the extent to which the variance in each subjective and objective variable was explained by the P300 component in Go trials, the P300 in No-go trials, and the dichotomous stimulation type “as a whole”, and second account for whether adding the P400 component in No-go trials as the fourth independent variable would improve regression precision. The Wilcoxon signed-rank test, paired-samples t-test, Shapiro–Wilk test, and hierarchical multiple regression analyses were conducted using IBM SPSS Statistics (version 26).

## 3. Results

### 3.1. Subjective Experience

**Figure 2:**
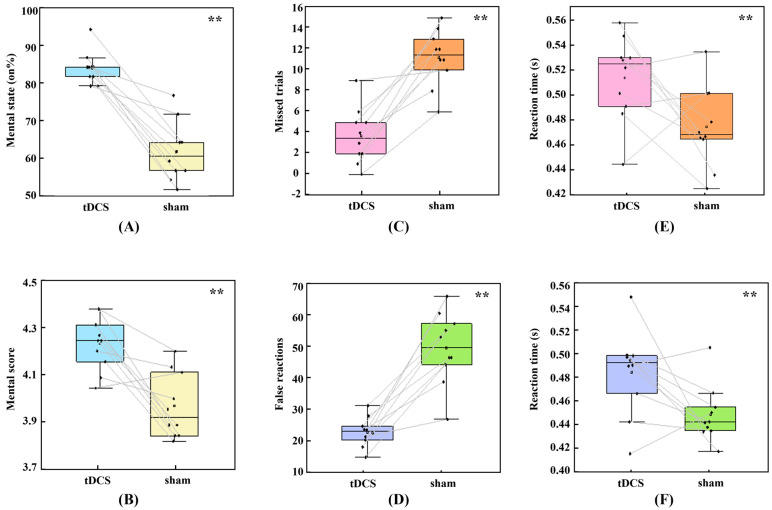
Subjective report results and behavioral performance after real high-definition transcranial direct current stimulation (HD-tDCS) and sham stimulation. (**A**) Percentage of “on the task” options selected in the total mental feedback; (**B**) averaged mental scores; (**C**) missed trials in Go trials; (**D**) false reactions in No-go trials; (**E**) averaged reaction time (RT) in the correct Go trials; and (**F**) averaged RT in the incorrect No-go trials. ** indicates a significant difference (*p* < 0.05) in the Wilcoxon signed-rank tests.

The 3D GNG tasks require participants to maintain their attention in the 3D VE; however, participant-declared attention is not always focused on the current 3D task. We compared the ratio of “on the task” option selection and averaged attentional scores in subjective answers between the real and sham stimulation conditions using Wilcoxon signed-rank tests. As illustrated in Figure 2A,B, there was a significant increase in selection of the “on the task” option (median [Mdn] = 23.750) when participants received real HD-tDCS over the rVLPFC compared to that in the sham condition, z = 2.81, *p* = 0.005. Additionally, the median mental score suggestively increased (Mdn = 0.363) in the real tDCS condition, z = 2.65, *p* = 0.008. Both these results demonstrated that anodal HD-tDCS could improve participant-perceived sustained attention.

### 3.2. Behavioral Performance and HDDM Metrics

Attentional status in VR could also be reflected in behavioral accuracy and RT in the 3D GNG tasks. As shown in Figure 2C–F, Wilcoxon signed-rank test analysis showed that, in the real HD-tDCS condition, missed trials decreased (Mdn = 8.000), z = −2.81, *p* = 0.005; false reactions decreased (Mdn = 25.500), z = −2.80, *p* = 0.005; RT in Go trials increased (Mdn = 0.055), z = 1.99, *p* = 0.047; and RT in No-go trials increased (Mdn = 0.036), z = 2.09, *p* = 0.037. Accordingly, anodal stimulation improved accuracy and slowed down responses in both Go and No-go trials.

**Figure 3:**
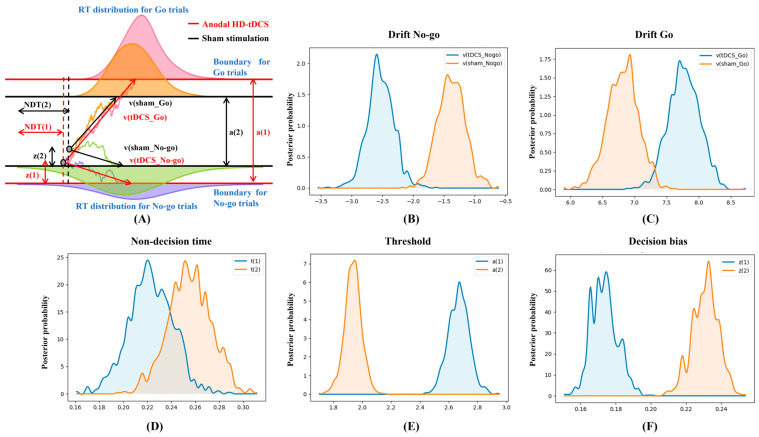
The hierarchical drift-diffusion model (HDDM) results after high-definition transcranial direct current stimulation (HD-tDCS) and sham stimulation. (**A**) Reaction time (RT) distributions and modeled streams of noisy observations, where the lengths of HDDM indicators are proportional to the averaged results of all participants after HD-tDCS and sham stimulation; (**B**) drift rates in No-go trials; (**C**) drift rates in Go trials; (**D**) non-decision time; (**E**) decision thresholds; and (**F**) initial decision bias.

Next, the underlying decision dynamics of behavioral performance were decomposed and represented using Bayesian estimation and the drift-diffusion model. Figure 3 displayed the contrasts of HDDM parameter distributions: v_nogo_sham_ > v_nogo_tDCS_ (*p* = 0.999), v_go_tDCS_ > v_go_sham_ (*p* = 0.997), NDT_sham_ > NDT_tDCS_ (*p* = 0.901), a_tDCS_ > a_sham_ (*p* = 1.000), and z_sham_ > z_tDCS_ (*p* = 1.000), where *p* is the probability of Bayesian estimation. Therefore, anodal HD-tDCS accelerated the speeds to characterize information in both Go and No-go trials, reduced the decision-preparation time, augmented the evidence to be accumulated, and decreased the initial tendency of pressing the spacebar with a great probability. Additionally, good convergence of MCMC (Supplementary Figure S2) agreed with the parameters sampled from the posterior distribution.

### 3.3. ERP Components

**Figure 4:**
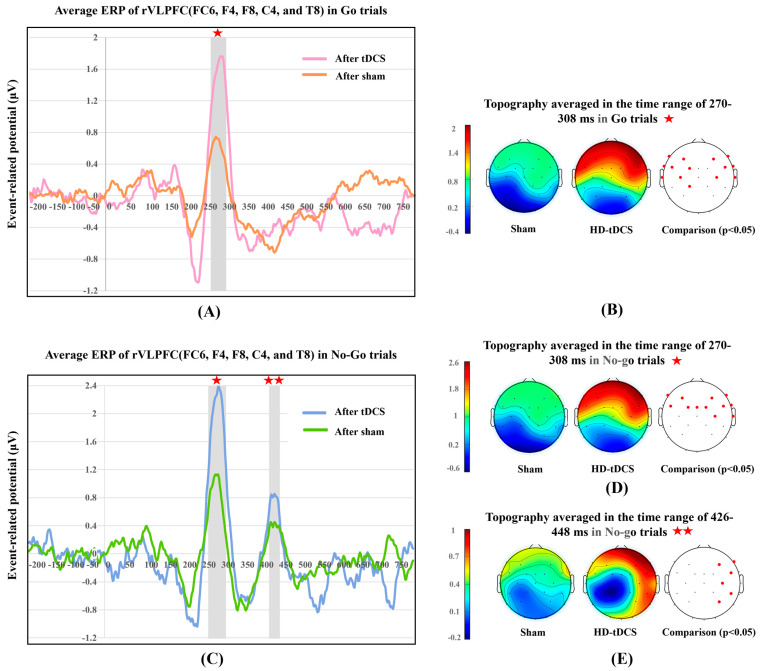
Contrasts of event-related potential (ERP) waveforms and scalp energy topographies. (**A**) ERP of Go trials averaged across the FC6, F4, F8, C4, and T8 channels; (**B**) topographies in Go trials and permutation comparisons of scalp energy in the P300 window; (**C**) ERP of No-go trials averaged across the FC6, F4, F8, C4, and T8 channels; (**D**) topographies in No-go trials and permutation comparisons of scalp energy in the P300 window; and (**E**) topographies in No-go trials and permutation comparisons of scalp energy in the P400 window. For the permutation results, the red and black solid circles correspond to the *p*-value of the corresponding electrode being less than or higher than 0.05, respectively. ★ represents the time window of 270–308 ms. ★★ represents the time window of 426–448 ms.

To further explore the neural alterations induced by HD-tDCS over the rVLPFC for both Go and No-go trials, we represented the continuous neural discharge patterns in the rVLPFC, and scalp topographies during the time frames of robust ERP components. Figure 4 depicts the neural difference between the real tDCS and sham stimulation conditions.

#### 3.3.1. P300 in Go and No-Go Trials

Considering both kinds of trials, as illustrated in Figure 4A–D, the P300 component was evoked in a 270–308 ms latency with a frontal positivity lateralized to the rVLPFC. For Go trials, HD-tDCS induced a significant P300 increase (mean [M] = 0.931; 95% confidence interval [CI]: 0.837–1.024), t(9) = 22.543, *p* < 0.001, d = 7.13, whose normality of group difference was not violated, *p* = 0.909. For No-go trials, electrical stimulation significantly increased the P300 amplitude (M = 1.167, 95% CI: 1.038–1.295), t(9) = 20.551, *p* < 0.001, d = 6.50, and here too the normality assumption was proved, *p* = 0.934. Permutation-based statistical analysis verified that the real stimulation significantly energized the frontal lobe in both Go (*p* < 0.05 for the F7, F3, F4, F8, FT9, FC5, FC1, FC6, FT10, T7, C3, C4, T8, and CP1 frontal electrodes) and No-go trials (*p* < 0.05 for the F7, F3, F4, F8, FC5, FC1, Fz, FC2, FC6, C4, and T8 frontal electrodes). Thus, real stimulation over the rVLPFC enhanced the target region neural arousal over the time frame of the P300 component for both Go and No-go trials.

#### 3.3.2. P400 in No-Go Trials

Figure 4C,E showed another robust component (P400) in No-go trials. The time window for this component was 426–448 ms, and scalp energy was found to be transformed from subtle activation of the frontal lobe in the sham condition to positivity focused in the right frontal and occipitotemporal (also called the fusiform gyrus) cortices under real stimulation (Figure 4E). Paired-samples t-test results showed that tDCS induced significantly higher P400 amplitudes (M = 0.392, 95% CI: 0.287–0.497), t(9) = 8.421, *p* < 0.001, d = 2.66, whose normality was evaluated as *p* = 0.164. Permutation testing verified that there was significant stimulation-elicited activation in the rVLPFC and fusiform gyrus (*p* < 0.05 for the F4, F8, FC6, C4, CP6, and P4 right cortical electrodes). Accordingly, HD-tDCS over the rVLPFC also promoted the concentrated neural discharges in the time window of the P400 component only in No-go trials.

### 3.4. Hierarchical Multiple Regression

Although HD-tDCS significantly altered subjective reports, objective outcomes, and physiological recordings, the correlations of ERP amplitudes with self-attentional perception and behavioral performance were not yet clarified. Therefore, we performed hierarchical multiple regression to sequentially explore the functional signatures of the different ERP components. As displayed in Table 1, the averaged P300 in Go trials, P300 in No-go trials, and stimulation being real or sham could conjunctively predict the following variables with significance: the proportion of selected “on the task” options (adjusted R^2^ = 0.755, F(3, 16) = 20.537, *p_model_* < 0.001), averaged mental score (adjusted R^2^ = 0.569, F(3, 16) = 9.359, *p_model_* < 0.001), total accuracy of all trials (adjusted R^2^ = 0.825, F(3, 16) = 30.873, *p_model_* < 0.001), drift of Go trials (adjusted R^2^ = 0.520, F(3, 16) = 3.987, *p_model_* = 0.027), drift of No-go trials (adjusted R^2^ = 0.865, F(3, 16) = 41.544, *p_model_* < 0.001), decision threshold (adjusted R^2^ = 0.918, F(3, 16) = 72.244, *p_model_* < 0.001), and decision bias (adjusted R^2^ = 0.996, F(3, 16) = 1512.664, *p_model_* < 0.001). Subsequent addition of the P400 component in No-go trials as the fourth predictor in the regression model did not increase the coefficient of determination significantly, despite the regression equation at the second hierarchical level remaining significant for the above independent variables. Moreover, the joint independent variables were not significantly related to RT (*p_model_* = 0.053) or non-decision time (*p_model_* = 0.157) even in the first hierarchical level of multiple regressions.

## 4. Discussion

According to the mainstream view, the next generation of the internet ecosystem will be the Metaverse, a set of interconnected virtual worlds in virtual, augmented, and mixed reality [50]. However, due to technical limitations, the omnipresent mind wandering occurs more frequently in ultrarealistic computer-simulated worlds [51]. This study addressed this challenge from a novel perspective involving brain stimulation and took a comprehensive model to explore sustained 3D attention. We applied HD-tDCS over the rVLPFC to prolong sustained attention in VE and evaluated the offline effects of stimulation; specifically, we tracked subjective mentality, objective manifestations, and neuroinformatics data during the developed 3D GNG task. Specific and significant stimulus-derived alterations were observed in each variable of these three aspects; furthermore, the neuro-mechanism underlying behavioral dynamics was also elucidated by the statistical links between the endogenous ERP components and exterior performance outcomes.

### 4.1. Methodology of Embodying and Motivating 3D Sustained Attention

To explore the mind-wandering situation in a simulated world, we developed a transformative GNG task in VR using customized scripts. The prototype used for the 3D GNG task is a classic 2D continuous mental paradigm for investigating reaction inhibition, which requires strenuous monitoring of the infrequent stop stimuli and deliberate withholding of the pre-potent response tendency [52]. Exact inhibition functions as a core element of self-control, supports flexible goal-directed behaviors, and reflects the efforts to maintain attention in an ever-changing environment [53]. In this regard, we argue that recordings of different natures in the 3D inhibitory task can reflect the neural attentional rhythms that oscillate between the external, ultrarealistic VR world and inner mentality.

The decision-making process, supported by multiple frontal regions, is fundamental to the 2D GNG task. Specifically, the anterior cingulate cortex could monitor the conflict caused by incorrect response impulse that contradicts actual trial information, and prevent interference from task-irrelevant cues [54,55,56]; the medial frontal cortex functions as error detection in hand-motion [57]; and the rVLPFC is associated with timely capture of important cues, inhibitory execution, and motion update [58,59]. Neuroimaging study has revealed that patients with rVLPFC lesions possess impaired inhibitory control, suggesting a unique status for rVLPFC in the traditional 2D GNG task; hence, it is inferred that neural arousal in this region may be crucial for the inhibitory control flows in the 3D VE as well [47,60,61,62]. To scrutinize this inference, we focally activated the right inferior frontal gyrus using anodal 4 × 1 HD-tDCS montages, wherein electrodes were attached only to the target cortical region, and compared the activation effects with those in the sham condition. In comparison, in the previously used two-sponge stimulation, the cathode was typically located in the target contralateral cortex (FC5), which would suppress the neuronal discharge of Broca’s area and eventually interfere with the experimental consequences of functional changes in the rVLPFC [23,63,64]. Thus, our arrangement could overcome the drawbacks of the traditional stimulation method and maximize the reliability of the neural effects being exactly attributable to the modulation of the rVLPFC.

### 4.2. Comprehensive Evidence for Extended Attention in VR

The results regarding external objective behaviors and perceived attention presented here support the hypothesis that tDCS modulates the performance of users in the 3D VE. Brunyé et al. found that electrical stimulation over the temporal lobe enhanced the 3D virtual navigation efficiency of individuals with a relatively weaker sense of direction [65]. Furthermore, Wolfgang et al. reported that tDCS over the right dorsolateral prefrontal cortex promoted the subjective sense of presence in the VE [66]. Similarly, in this study, analysis of the questionnaire responses showed that stimulation significantly improved subjectively perceived 3D attention. Regarding superficial behavioral performance, activation of the rVLPFC could lead to greater accuracy and increased RTs in the virtual 3D environment. This seems to suggest that when rVLPFC neuron firing increases, humans trade speed for accuracy to make more prudent decisions [67]. To verify this possibility, we further represented the underlying decision-making dynamics using the HDDM. As illustrated in Figure 3A, in the stream of noisy observations under the stimulation condition, mind preparation time decreased, momentary evidence accumulated faster over time, the evidence boundary became broader, and a priori response inhibition increased. The former two transformations are supposed to correspond to a shorter RT, and the latter two should correlate with improved accuracy; however, RT was observed to increase, which was contrary to the theoretical conjecture. The reason for this increase in RT could be that the increased speed of collecting information could not break through the robust cover of the greater evidence threshold. Thus, increased accuracy in the 3D VE was not simply at the expense of slower decision speed, but also involved an underlying conflict between an increase in both speed and the evidence threshold. It has been reported that the attentional function is grounded on multiple functionally heterogeneous cortices, which constitute the sustained attention network [68]. Posner et al. separated the attention network into three components: alerting, orienting, and execution [69]. Specifically, alerting makes use of the thalamus, anterior, and posterior cortices to maintain the vigilance for the ambient environment; orienting attention activates the precentral gyrus and superior parietal sites to select excitability encoded in sensory system for use; and executive control of attention will motivate anterior cingulate, left, and right frontal areas to achieve conflict monitoring and inhibition [70]. The 3D GNG task-measured reaction inhibitions are regarded to mainly reflect the executive control of attention in the 3D VE [71]. Conversely, mind wandering is generally supported by the default-mode network [72]. Both networks could voluntarily activate and deactivate in specific time windows, but they are intrinsically anticorrelated in competing for neural resources towards the external tasks or internal mentality [68]. In this study, the rVLPFC served as a node of the executive attention network, and electrical stimulation on it might imply global activation of executing functional connectivity. Hereby the executive attention could win the competition against mind wandering to focus on the external 3D continuous inhibitory tasks, and inspire improved perceived attention, more prudent behavior, and a more robustly decomposed underlying decision process.

Furthermore, the ERP analysis allowed us to investigate whether and how tDCS affects the shifts of internal neuronal discharges over time during decisions in 3D VR [73]. In both Go and No-go trials, tDCS enhanced P300 amplitudes in the rVLPFC and induced frontal positivity, which was mildly lateralized to the rVLPFC. This confirms that electrical stimulation has an activation effect on rVLPFC and also that this regional ERP component could be the neural signature of 3D inhibitory tasks. The enhancements of P300 amplitudes in the rVLPFC might be the regional embody of the HD-tDCS-activated executing control of attention network with a fixed rhythm. A much-debated question is whether the third positive deflection distributed in the rVLPFC is associated solely with the inhibition of dominant motor responses to stop signals or with broader response evaluation whenever important cues appear [74,75]. In this study, we observed robust P300 peaks and obvious rVLPFC activation not only in No-go but also in Go trials, which supports the latter hypothesis, that is, this gyrus is actually responsible for overall cue processing in continuous 3D tasks. In addition, the topographies presented that strong scalp energy robustly distributed throughout the frontal lobe and not just the rVLPFC, proving that multiple frontal areas engaged in the intricate decision process of 3D GNG tasks as well.

Importantly, the regression models could demonstrate the correlations of neural patterns with subjective and objective variables, which further provides evidence for the physiologically effective mechanism of tDCS. Based on the variation tendency of each variable from sham to tDCS conditions discussed above, it can be inferred that, real tDCS (dichotomous stimulation type) and greater rVLPFC activation (higher P300 amplitudes in both Go and No-go trials) boost subjective experiences, accuracy, and decision threshold, prolong RT, and shorten decision-preparation time. Real tDCS and more activated rVLFPC (higher P300 amplitude) in Go or No-go trials accelerate evidence accumulation in the corresponding trials. Furthermore, real tDCS, greater rVLPFC arousal (higher P300 amplitude) in No-go trials, and lower rVLPFC arousal (smaller P300 amplitude) in Go trials, strengthen the initial motor inhibition. However, the signs of coefficients in regression prediction equations only fit the inferences for the drift rate of Go trials and the decision threshold. It should be highlighted that the construction of a regression model cannot simply refer to the significance level of the *p*-value but needs to judge from theoretical considerations [67]. While some of prediction equations here were significant, the alteration trends predicted by independent variables disagree with the interpretations of the dynamic mechanism underlying each dependent variable and thus are considered valueless. Nevertheless, the valid predictions of some of the HDDM decomposed indicators do support the view that stimuli-induced neural activations in the rVLPFC are the intrinsic factors affecting the observed phenomenological behavioral alterations. Previously, Wu et al. compared the inhibitory ability and the corresponding neural substrates between children with attention-deficit hyperactivity disorder (ADHD) and healthy controls by 2D GNG tasks and functional near-infrared spectroscopy, respectively [71]. Results showed that the prefrontal activation during GNG tasks was decreased for ADHD patients, but the behavioral performance was not significantly different between the two groups. The concrete links of attentional performance with underlying neural arousal remained unclear. Osimo et al. reported that the gray matter density of several frontal regions was positively related to the 2D GNG performance. This statistical correlation suggested that frontal morphology could directly influence attentional behaviors, with a great possibility to improve attention by neural modulation [76]. Silva et al. discovered that anodal electrical stimulation on the frontal lobe could effectively modulate the attentional orientation and execution, which was demonstrated in the behavioral scores in professional 2D tasks [77]. In this regard, the regression models here further explore that the highly focal neural activation in rVLPFC could facilitate 3D executing attention in the perspective of an underlying behavioral decision model, that is, decomposed HDDM indicators. 

Additionally, another evidently positive deflection in the 426–448 ms time window was only observed in No-go trials, which was significantly increased by HD-tDCS as well. In this time window, the positive energy was delivered to the frontal lobe in the sham condition but to the right frontal and fusiform cortices in the real tDCS condition. The concentration of energy in the rVLPFC under both conditions confirms that calculating mean P400 amplitudes in the FC6, F4, F8, C4, and T8 channels are proper manipulation. Khoshnoud et al. regarded the frontal ERP component at 400–600 ms as the second process of visual stimulus encoding [78]. Consistently, in the sham condition, the distribution of frontal energy at 426–448 ms may originate from the second confirmation of especially important cues, i.e., stop signals [79]. To detect whether the tDCS-improved P400 amplitudes involve better cue validation and external outcomes, we added the P400 component as the fourth regressor in the second hierarchical regression model. However, this additional regressor could not significantly improve the determination coefficient of any phenomenal performance variable, implying that P400 increase may not signify an increased allocation of neural resources to the second decision encoding. Some studies have suggested that positive arousal in the right fusiform gyrus at 400–600 ms may be associated with emotion regulation for novel visual stimuli [20,80]. Therefore, we speculate that the extra positive energy in the rVLPFC and fusiform gyrus in this study reflects another specific emotional signal processing pattern induced by electrical stimulation; in other words, rVLPFC impulses for second recognition of especially important cues flow into the fusiform gyrus to characterize the novelty evoked by infrequent visual trials. However, this neural pathway should be further investigated using functional magnetic resonance imaging to pinpoint the involved functional dipoles with high spatial resolution and confirm that they do not exist in the right fusiform gyrus when no electrical stimulation is applied.

### 4.3. Limitations

First, the 3D GNG tasks used as the sustained attention paradigm in this study are relatively undemanding and unitary, aiding the intensification of the signature of each variable and intuitively orchestrating diverse variables into a comprehensive model to test the attentional level. However, VR tasks in real-life are more complex and erratic; therefore, more naturalistic experimental setups are required to generalize our findings to the broader VR ecology. Second, the EEG signals of the FP1 and FP2 channels were excluded from our analyses due to high background noise. However, the remaining 10 frontal electrodes, which account for over 30% of the total number of electrodes, are considered to be able to robustly localize the frontal signal source. Other smaller VR headsets should be considered to avoid sacrificing data from the excluded EEG electrodes. Third, the significant *p*-values and large effects were detected in a small sample size of 10 participants, which makes the results less credible. In the case of limited subjects, we adopted a within-subjects design to minimize the interference caused by inter-subject variability and effectively determine intervention effects [81]. Nevertheless, larger groups of participants should be convened to further verify the conclusions stated in this study. Lastly, a neural pathway between the rVLPFC and fusiform gyrus is assumed to represent a novelty emotion evoked by infrequent visual cues. Though we provide some insights for neural interactions in the 3D GNG tasks, this tentative hypothesis should be further validated by more rigorous experimental designs and neural models.

## 5. Conclusions

The findings of this study preliminarily revealed that HD-tDCS over the rVLPFC promoted perceived attention, suppressed hasty reactions, facilitated a robust decision-making process, and intensified rVLPFC arousal in 3D GNG tasks. From the neurophysiological perspective, we investigated regional cortical arousal, which was further validated to be intuitively and effectively linked with the decomposed behavioral variables. Therefore, we propose that this experimental approach can ameliorate mind wandering in VR and characterize 3D sustained attention to investigate the proximate effects of the electrical stimulation. The patterns that HD-tDCS inspired 3D attention discovered here could boost more widespread application of VR for brain–machine interfaces in real life. To better understand the impact of electrical modulation on the holistic attention networks, we will constitute the functional connectivity in future studies to clarify the intricate statistical dependencies among cortical regions in the 3D continuous paradigm.

## Figures and Tables

**Table 1:** Summary of hierarchical multiple regression results, where event-related potential components and stimulation type were the independent variables, and each subjective and objective result was the dependent variable.

Variables	Model 1 (Stimulation Type, P300 in No-Go, P300 in Go)		Model 2 (P400 in No-Go Added)	
	Adjusted R^2^	Regression Prediction Equation	Variation of Adjusted R^2^	Regression Prediction Equation
Mental state	0.755 **	y = 67 + 25.43x_1_ − 16.29x_2_ + 17x_3_ **	−0.011	y = 69.52 + 30.04x_1_ − 29.1x_2_ + 21.34x_3_ + 16.06x_4_ **
Mental score	0.569 **	y = 3.68 − 0.16x_1_ − 0.26x_2_ + 0.8x_3_ **	−0.026	y = 3.7 − 0.1x_1_ − 0.41x_2_ + 0.86x_3_ + 0.19x_4_ *
Accuracy	0.825 **	y = 0.84 + 0.05x_1_ − 0.05x_2_ + 0.09x_3_ **	−0.011	y = 0.84 + 0.05x_1_ − 0.05x_2_ + 0.09x_3_ + 0.01x_4_ **
RT	0.255	y = 0.54 + 0.15x_1_ − 0.01x_2_ − 0.11x_3_	0.049	y = 0.54 + 0.15x_1_ − 0.01x_2_ − 0.11x_3_ − 0.01x_4_
v_go_	0.520 *	y = 8.69 + 2.95x_1_ − 3.68x_2_ + 2.47x_3_ *	0.026	y = 9.34 + 4.16x_1_ − 7.04x_2_ + 3.61x_3_ + 4.21x_4_ *
v_nogo_	0.865 **	y = −1.81 − 1.61x_1_ + 0.73x_2_ − 0.41x_3_ **	−0.009	y = −1.83 − 1.66x_1_ + 0.86x_2_ − 0.46x_3_ − 0.16x_4_ **
NDT	0.134	y = 0.45 + 0.23x_1_ − 0.14x_2_ − 0.11x_3_	0.110	y = 0.48 + 0.28x_1_ − 0.26x_2_ − 0.07x_3_ + 0.16x_4_
a	0.918 **	y = 1.74 + 0.44x_1_ + 0.02x_2_ + 0.29x_3_ **	0.001	y = 1.66 + 0.29x_1_ + 0.43x_2_ + 0.15x_3_ − 0.52x_4_ **
z	1.00 **	y = 0.23 − 0.06x_1_ + 0.01x_2_ − 0.01x_3_ **	0	y = 0.23 − 0.06x_1_ + 0.01x_2_ − 0.01x_3_ − 0.01x_4_ **

Adjusted R^2^, the coefficient of determination correcting a positive bias, is the proportion of variance in the dependent variable that is explained by the independent variables, and is also an estimate of effect size; variation of adjusted R^2^, the value of adjusted R^2^ in the second hierarchy minus the adjusted R^2^ in the first hierarchy; y, the corresponding dependent variable; x_1_, the first dichotomous independent variable (stimulation type), where the value 1 corresponds to real high-definition transcranial direct current stimulation and the value 0 corresponds to sham stimulation; x_2_, the second continuous independent variable (P300 amplitude in No-go trials); x_3_, the third continuous independent variable (P300 amplitude in Go trials); x_4_, the fourth continuous independent variable (P400 amplitude in No-go trials); * *p* < 0.05. ** *p* < 0.001. RT, reaction time. v_go_, drift rate of Go trials. v_nogo_, drift rate of No-go trials. NDT, non-decision time. a, decision threshold. z, initial decision bias.

## Data Availability

The data presented in this study are available on request from the corresponding author. The data are not publicly available due to institutional review board restrictions.

## Supplementary Materials

The following supporting information can be downloaded at https://www.mdpi.com/article/10.3390/bioengineering10060721/s1, Brief training; Figure S1: The collapsed localizer of ERP waveforms in the rVLPFC for the Go trials and No-go trials and Figure S2: The trace, autocorrelation, and marginal posterior of each HDDM parameter.

## References

[1] Xiong J., Hsiang E.-L., He Z., Zhan T., Wu S.-T. (2021). Augmented reality and virtual reality displays: Emerging technologies and future perspectives. Light Sci. Appl..

[2] Zhan T., Yin K., Xiong J., He Z., Wu S.-T. (2020). Augmented reality and virtual reality displays: Perspectives and challenges. iScience.

[3] Qiao J., Li X., Wang Y., Wang Y., Li G., Lu P., Wang S. (2022). The infraslow frequency oscillatory transcranial direct current stimulation over the left dorsolateral prefrontal cortex enhances sustained attention. Front. Aging Neurosci..

[4] Andrillon T., Burns A., Mackay T., Windt J., Tsuchiya N. (2021). Predicting lapses of attention with sleep-like slow waves. Nat. Commun..

[5] Litleskare S. (2021). The relationship between postural stability and cybersickness: It’s complicated–an experimental trial assessing practical implications of cybersickness etiology. Physiol. Behav..

[6] Nie G.-Y., Duh H.B.-L., Liu Y., Wang Y. (2019). Analysis on mitigation of visually induced motion sickness by applying dynamical blurring on a user’s retina. IEEE Trans. Vis. Comput. Graph..

[7] Fernandes A.S., Feiner S.K. Combating VR sickness through subtle dynamic field-of-view modification. Proceedings of the 2016 IEEE Symposium on 3D User Interfaces (3DUI).

[8] Lin J.-W., Duh H.B.-L., Parker D.E., Abi-Rached H., Furness T.A. Effects of field of view on presence, enjoyment, memory, and simulator sickness in a virtual environment. Proceedings of the IEEE Virtual Reality 2002.

[9] Thair H., Holloway A.L., Newport R., Smith A.D. (2017). Transcranial direct current stimulation (tDCS): A beginner’s guide for design and implementation. Front. Neurosci..

[10] Zhao H., Qiao L., Fan D., Zhang S., Turel O., Li Y., Li J., Xue G., Chen A., He Q. (2017). Modulation of brain activity with noninvasive transcranial direct current stimulation (tDCS): Clinical applications and safety concerns. Front. Psychol..

[11] Mashour G.A., Pal D., Brown E.N. (2022). Prefrontal cortex as a key node in arousal circuitry. Trends Neurosci..

[12] Takahashi M., Ikegami M. (2008). Differential Frontal Activation during Exogenous and Endogenous Orientation of Visuospatial Attention. Neuropsychobiology.

[13] Levy B.J., Wagner A.D. (2011). Cognitive control and right ventrolateral prefrontal cortex: Reflexive reorienting, motor inhibition, and action updating. Ann. N. Y. Acad. Sci..

[14] Corbetta M., Shulman G.L. (2002). Control of goal-directed and stimulus-driven attention in the brain. Nat. Rev. Neurosci..

[15] Gibson B.C., Mullins T.S., Heinrich M.D., Witkiewitz K., Alfred B.Y., Hansberger J.T., Clark V.P. (2020). Transcranial direct current stimulation facilitates category learning. Brain Stimul..

[16] Coffman B.A., Trumbo M.C., Clark V.P. (2012). Enhancement of object detection with transcranial direct current stimulation is associated with increased attention. BMC Neurosci..

[17] Campanella S., Schroder E., Monnart A., Vanderhasselt M.-A., Duprat R., Rabijns M., Kornreich C., Verbanck P., Baeken C. (2017). Transcranial direct current stimulation over the right frontal inferior cortex decreases neural activity needed to achieve inhibition: A double-blind ERP study in a male population. Clin. EEG Neurosci..

[18] Hampshire A., Chamberlain S.R., Monti M.M., Duncan J., Owen A.M. (2010). The role of the right inferior frontal gyrus: Inhibition and attentional control. Neuroimage.

[19] Thomas C., Ghodratitoostani I., Delbem A.C., Ali A., Datta A. Influence of gender-related differences in transcranial direct current stimulation: A Computational Study. Proceedings of the 2019 41st Annual International Conference of the IEEE Engineering in Medicine and biology Society (EMBC).

[20] Cohen J. (2013). Statistical Power Analysis for the Behavioral Sciences.

[21] Villamar M.F., Wivatvongvana P., Patumanond J., Bikson M., Truong D.Q., Datta A., Fregni F. (2013). Focal modulation of the primary motor cortex in fibromyalgia using 4 × 1-ring high-definition transcranial direct current stimulation (HD-tDCS): Immediate and delayed analgesic effects of cathodal and anodal stimulation. J. Pain.

[22] Ruch S., Fehér K., Homan S., Morishima Y., Mueller S.M., Mueller S.V., Dierks T., Grieder M. (2021). Bi-temporal anodal transcranial direct current stimulation during slow-wave sleep boosts slow-wave density but not memory consolidation. Brain Sci..

[23] Sreeraj V.S., Dinakaran D., Parlikar R., Chhabra H., Selvaraj S., Shivakumar V., Bose A., Narayanaswamy J.C., Venkatasubramanian G. (2018). High-definition transcranial direct current simulation (HD-tDCS) for persistent auditory hallucinations in schizophrenia. Asian J. Psychiatry.

[24] Friedman N.P., Robbins T.W. (2022). The role of prefrontal cortex in cognitive control and executive function. Neuropsychopharmacology.

[25] Kessler S.K., Turkeltaub P.E., Benson J.G., Hamilton R.H. (2012). Differences in the experience of active and sham transcranial direct current stimulation. Brain Stimul..

[26] Nitsche M., Fricke K., Henschke U., Schlitterlau A., Liebetanz D., Lang N., Henning S., Tergau F., Paulus W. (2003). Pharmacological modulation of cortical excitability shifts induced by transcranial direct current stimulation in humans. J. Physiol..

[27] Nitsche M.A., Paulus W. (2001). Sustained excitability elevations induced by transcranial DC motor cortex stimulation in humans. Neurology.

[28] Hays J., Wong C., Soto F.A. (2020). FaReT: A free and open-source toolkit of three-dimensional models and software to study face perception. Behav. Res. Methods.

[29] O’Callaghan C., Hall J.M., Tomassini A., Muller A.J., Walpola I.C., Moustafa A.A., Shine J.M., Lewis S.J. (2017). Visual hallucinations are characterized by impaired sensory evidence accumulation: Insights from hierarchical drift diffusion modeling in Parkinson’s disease. Biol. Psychiatry Cogn. Neurosci. Neuroimaging.

[30] Turner B.M., Van Maanen L., Forstmann B.U. (2015). Informing cognitive abstractions through neuroimaging: The neural drift diffusion model. Psychol. Rev..

[31] Wiecki T.V., Sofer I., Frank M.J. (2013). HDDM: Hierarchical Bayesian estimation of the drift-diffusion model in Python. Front. Neuroinformatics.

[32] Zhang J., Rowe J.B. (2014). Dissociable mechanisms of speed-accuracy tradeoff during visual perceptual learning are revealed by a hierarchical drift-diffusion model. Front. Neurosci..

[33] Wang Z.-J., Kim E.-S., Noh B.H., Liang J.-G., Lee D., Hur Y.J., Kim N.-Y., Kim H.-D. (2020). Alteration in brain connectivity in patients with Dravet syndrome after vagus nerve stimulation (VNS): Exploration of its effectiveness using graph theory analysis with electroencephalography. J. Neural Eng..

[34] Delorme A., Makeig S. (2004). EEGLAB: An open source toolbox for analysis of single-trial EEG dynamics including independent component analysis. J. Neurosci. Methods.

[35] Lopez-Calderon J., Luck S.J. (2014). ERPLAB: An open-source toolbox for the analysis of event-related potentials. Front. Hum. Neurosci..

[36] Read G.L., Innis I.J. (2017). Electroencephalography (Eeg). Int. Encycl. Commun. Res. Methods.

[37] Li Y., Wang Y., Zhang B., Wang Y., Zhou X. (2018). Electrophysiological responses to expectancy violations in semantic and gambling tasks: A comparison of different EEG reference approaches. Front. Neurosci..

[38] Sander T., Bock A., Leistner S., Kühn A., Trahms L. Coherence and imaginary part of coherency identifies cortico-muscular and cortico-thalamic coupling. Proceedings of the 2010 Annual International Conference of the IEEE Engineering in Medicine and Biology.

[39] Pavone E.F., Tieri G., Rizza G., Tidoni E., Grisoni L., Aglioti S.M. (2016). Embodying others in immersive virtual reality: Electro-cortical signatures of monitoring the errors in the actions of an avatar seen from a first-person perspective. J. Neurosci..

[40] Wang Z.J., Noh B.H., Kim E.S., Yang D., Yang S., Kim N.Y., Hur Y.J., Kim H.D. (2022). Brain network analysis of interictal epileptiform discharges from ECoG to identify epileptogenic zone in pediatric patients with epilepsy and focal cortical dysplasia type II: A retrospective study. Front. Neurol..

[41] Makeig S., Bell A., Jung T.-P., Sejnowski T.J. (1995). Independent component analysis of electroencephalographic data. Adv. Neural Inf. Process. Syst..

[42] Pion-Tonachini L., Kreutz-Delgado K., Makeig S. (2019). ICLabel: An automated electroencephalographic independent component classifier, dataset, and website. NeuroImage.

[43] Lin C.-T., Chung I.-F., Ko L.-W., Chen Y.-C., Liang S.-F., Duann J.-R. (2007). EEG-based assessment of driver cognitive responses in a dynamic virtual-reality driving environment. IEEE Trans. Biomed. Eng..

[44] Deng Z., Zhang Z. (2014). Event-related complexity analysis and its application in the detection of facial attractiveness. Int. J. Neural Syst..

[45] Logan G.D., Cowan W.B. (1984). On the ability to inhibit thought and action: A theory of an act of control. Psychol. Rev..

[46] Friedrich J., Beste C. (2018). Paradoxical, causal effects of sensory gain modulation on motor inhibitory control—A tDCS, EEG-source localization study. Sci. Rep..

[47] Dubreuil-Vall L., Gomez-Bernal F., Villegas A.C., Cirillo P., Surman C., Ruffini G., Widge A.S., Camprodon J.A. (2021). Transcranial direct current stimulation to the left dorsolateral prefrontal cortex improves cognitive control in patients with attention-deficit/hyperactivity disorder: A randomized behavioral and neurophysiological study. Biol. Psychiatry Cogn. Neurosci. Neuroimaging.

[48] Luck S.J., Gaspelin N. (2017). How to get statistically significant effects in any ERP experiment (and why you shouldn’t). Psychophysiology.

[49] Clayson P.E., Baldwin S.A., Larson M.J. (2013). How does noise affect amplitude and latency measurement of event-related potentials (ERPs)? A methodological critique and simulation study. Psychophysiology.

[50] Huynh-The T., Pham Q.-V., Pham X.-Q., Nguyen T.T., Han Z., Kim D.-S. (2023). Artificial intelligence for the metaverse: A survey. Eng. Appl. Artif. Intell..

[51] Zhang J., Zhou H., Geng F., Song X., Hu Y. (2021). Internet gaming disorder increases mind-wandering in young adults. Front. Psychol..

[52] Garavan H., Ross T.J., Murphy K., Roche R.A., Stein E.A. (2002). Dissociable executive functions in the dynamic control of behavior: Inhibition, error detection, and correction. Neuroimage.

[53] Aron A.R., Robbins T.W., Poldrack R.A. (2014). Inhibition and the right inferior frontal cortex: One decade on. Trends Cogn. Sci..

[54] Nieuwenhuis S., Yeung N., Van Den Wildenberg W., Ridderinkhof K.R. (2003). Electrophysiological correlates of anterior cingulate function in a go/no-go task: Effects of response conflict and trial type frequency. Cogn. Affect. Behav. Neurosci..

[55] Koizumi A., Lau H., Shimada Y., Kondo H.M. (2018). The effects of neurochemical balance in the anterior cingulate cortex and dorsolateral prefrontal cortex on volitional control under irrelevant distraction. Conscious. Cogn..

[56] Schulz K.P., Bédard A.C.V., Czarnecki R., Fan J. (2011). Preparatory activity and connectivity in dorsal anterior cingulate cortex for cognitive control. Neuroimage.

[57] Rodrigo A.H., Di Domenico S.I., Ayaz H., Gulrajani S., Lam J., Ruocco A.C. (2014). Differentiating functions of the lateral and medial prefrontal cortex in motor response inhibition. Neuroimage.

[58] Chikazoe J. (2010). Localizing performance of go/no-go tasks to prefrontal cortical subregions. Curr. Opin. Psychiatry.

[59] Liddle P.F., Kiehl K.A., Smith A.M. (2001). Event-related fMRI study of response inhibition. Hum. Brain Mapp..

[60] Aron A.R., Fletcher P.C., Bullmore E.T., Sahakian B.J., Robbins T.W. (2003). Stop-signal inhibition disrupted by damage to right inferior frontal gyrus in humans. Nat. Neurosci..

[61] Rubia K., Smith A.B., Brammer M.J., Taylor E. (2003). Right inferior prefrontal cortex mediates response inhibition while mesial prefrontal cortex is responsible for error detection. Neuroimage.

[62] Lapenta O.M., Di Sierve K., de Macedo E.C., Fregni F., Boggio P.S. (2014). Transcranial direct current stimulation modulates ERP-indexed inhibitory control and reduces food consumption. Appetite.

[63] Reinhart R.M., Cosman J.D., Fukuda K., Woodman G.F. (2017). Using transcranial direct-current stimulation (tDCS) to understand cognitive processing. Atten. Percept. Psychophys..

[64] Hupfeld K.E., Ketcham C.J., Schneider H.D. (2017). Transcranial direct current stimulation (tDCS) to Broca’s area: Persisting effects on non-verbal motor behaviors. Neurol. Disord. Ther..

[65] Brunyé T.T., Holmes A., Cantelon J., Eddy M.D., Gardony A.L., Mahoney C.R., Taylor H.A. (2014). Direct current brain stimulation enhances navigation efficiency in individuals with low spatial sense of direction. Neuroreport.

[66] Kogler W., Wood G., Kober S.E. (2021). Effects of electrical brain stimulation on brain indices and presence experience in immersive, interactive virtual reality. Virtual Real..

[67] Fox J. (2019). Regression Diagnostics: An Introduction.

[68] Pamplona G.S., Heldner J., Langner R., Koush Y., Michels L., Ionta S., Scharnowski F., Salmon C.E. (2020). Network-based fMRI-neurofeedback training of sustained attention. Neuroimage.

[69] Posner M.I., Petersen S.E. (1990). The attention system of the human brain. Annu. Rev. Neurosci..

[70] Posner M.I., Rothbart M.K., Voelker P. (2016). Developing brain networks of attention. Curr. Opin. Pediatr..

[71] Wu T., Liu X., Cheng F., Wang S., Li C., Zhou D., Zhang W. (2023). Dorsolateral prefrontal cortex dysfunction caused by a go/no-go task in children with attention-deficit hyperactivity disorder: A functional near-infrared spectroscopy study. Front. Neurosci..

[72] Mason M.F., Norton M.I., Van Horn J.D., Wegner D.M., Grafton S.T., Macrae C.N. (2007). Wandering minds: The default net-work and stimulus-independent thought. Science.

[73] Keil A., Debener S., Gratton G., Junghöfer M., Kappenman E.S., Luck S.J., Luu P., Miller G.A., Yee C.M. (2014). Committee report: Publication guidelines and recommendations for studies using electroencephalography and magnetoencephalography. Psychophysiology.

[74] Wessel J.R., Aron A.R. (2015). It’s not too late: The onset of the frontocentral P 3 indexes successful response inhibition in the stop-signal paradigm. Psychophysiology.

[75] Huster R.J., Enriquez-Geppert S., Lavallee C.F., Falkenstein M., Herrmann C.S. (2013). Electroencephalography of response inhibition tasks: Functional networks and cognitive contributions. Int. J. Psychophysiol..

[76] Osimo S.A., Piretti L., Ionta S., Rumiati R.I., Aiello M. (2021). The neural substrates of subliminal attentional bias and reduced inhibition in individuals with a higher BMI: A VBM and resting state connectivity study. NeuroImage.

[77] Silva A.F., Zortea M., Carvalho S., Leite J., Torres I.L.D.S., Fregni F., Caumo W. (2017). Anodal transcranial direct current stimulation over the left dorsolateral prefrontal cortex modulates attention and pain in fibromyalgia: Randomized clinical trial. Sci. Rep..

[78] Khoshnoud S., Shamsi M., Nazari M.A., Makeig S. (2018). Different cortical source activation patterns in children with attention deficit hyperactivity disorder during a time reproduction task. J. Clin. Exp. Neuropsychol..

[79] Bhavnani S., Lockwood Estrin G., Haartsen R., Jensen S.K., Gliga T., Patel V., Johnson M.H. (2021). EEG signatures of cognitive and social development of preschool children—A systematic review. PLoS ONE.

[80] Zhang M., Tian F., Wu X., Liao S., Qiu J. (2011). The neural correlates of insight in Chinese verbal problems: An event related-potential study. Brain Res. Bull..

[81] Lisanby S.H., Luber B., Schlaepfer T.E., Sackeim H.A. (2003). Safety and feasibility of magnetic seizure therapy (MST) in major depression: Randomized within-subject comparison with electroconvulsive therapy. Neuropsychopharmacology.

