# Supplementary material for: High-Definition Transcranial Direct Current Stimulation in the Right Ventrolateral Prefrontal Cortex Lengthens Sustained Attention in Virtual Reality
Source: Bioengineering (Basel). 2023 Jun 14;10(6):721. doi: 10.3390/bioengineering10060721 (PMC10294866; doi:10.3390/bioengineering10060721)
Supplement: Supplementary file 1 [file bioengineering-10-00721-s001.zip › bioengineering-2411249-SM.pdf]

High-definition transcranial direct current stimulation in the ventrolateral prefrontal cortex lengthens sustained attention in virtual reality

Shan Yang[1,2], Ganbold Enkhzaya[1,2], Bao-Hua Zhu[1], Jian Chen[1], Zhi-Ji Wang[1,3,*], Eun-Seong Kim[1,*], and Nam-Young Kim[1,2,*]

[1] RFIC Center, Department of Electronic Engineering, Kwangwoon University, Nonwon-gu, Seoul, South Korea

[2] NDAC Center, Kwangwoon University, Nonwon-gu, Seoul, South Korea

[3] Department of Pediatrics, Severance Children's Hospital, Yonsei University, Republic of Korea

* Correspondence: zhiji-wang@hotmail.com (Z.J.W.), eskim@wavepia.com (E.S.K.), and nykim@kw.ac.kr (N.Y.K.)

Supplementary Methods

Brief training. During the pilot section (24 trials), every model of eight kinds of 3D face appeared three times. Each face was displayed for 1 s if subjects did not press spacebar; the face would disappear immediately if a response was detected. After every trial offline, feedback of 'right response' would be presented for 1 s if the response was right, whereas feedback of 'this is a sad face, you should press spacebar' or 'this is a happy face, you should not press spacebar' would be displayed for 3 s for the wrong response to Go and No-go trials, respectively. The interval between trials was fixed at 1.5 s.

Supplementary Figure S1

The collapsed localizer of ERP waveforms in the rVLPFC for the Go trials (A) and No-go trials (B)

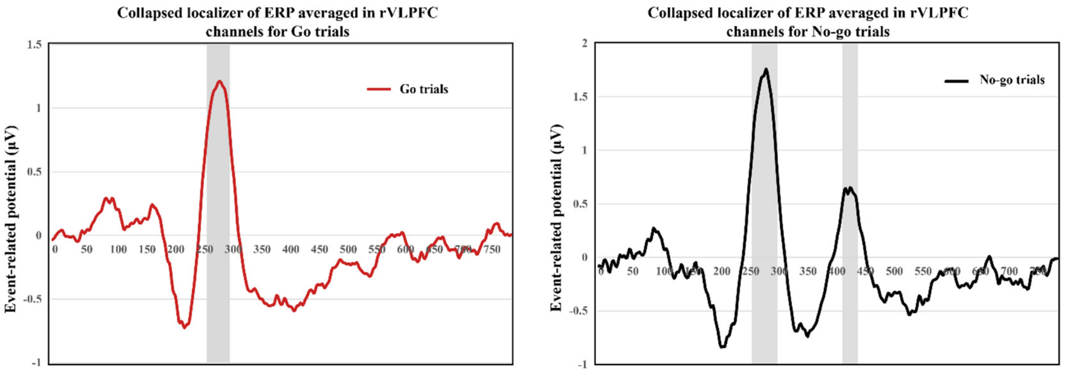

(A) (B)

Collapsed localizers of ERP. For both Go and No-go trials, we calculated the grand averaged ERP waveforms across the tDCS and sham conditions in all EEG channels. Subsequently, as illustrated in Figure S1, we averaged the collapsed ERP waveforms across channels FC6, F4, F8, C4, and T8. The 270–308 ms range was set as the P300 component time window for both Go and No-go trials by visual inspection. Additionally, in the collapsed waveform of No-go trials, another positive deflection at P400 was visually detected in the 426–448 ms range. Thus, in this study, we only focused on investigating the ERP P300 component in Go and No-go trials, and the P400 component in No-go trials.

Supplementary Figure S2

The trace (upper left corner), autocorrelation (lower left corner), and marginal posterior (right plot) of each HDDM parameter—the drift rates of Go trials after tDCS, Go trials after sham stimulation, No-go trials after tDCS, and No-go trials after sham stimulation; the non-decision times after tDCS and after sham stimulation; the decision thresholds after tDCS and after sham stimulation; and the initial decision bias after tDCS and after sham stimulation. Geweke's statistic for each parameter was printed out to represent the result of comparing the average and variance values of the beginning and ending segments of the chain, where 'true' means no statistically significant difference and 'false' means a statistically significant difference.

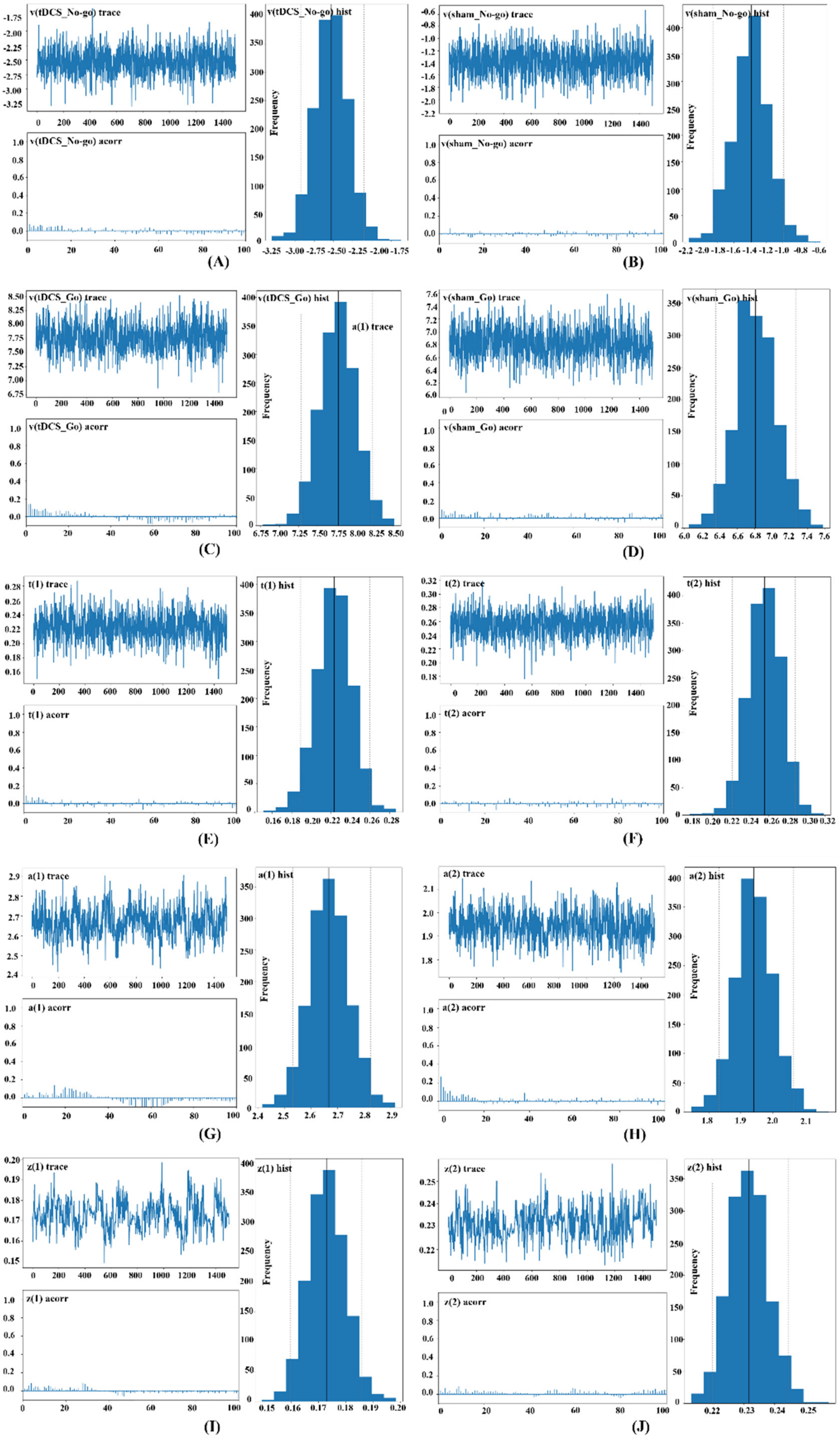
v(tDCS_No-go) trace
v(tDCS_No-go) acorr
v(tDCS_No-go) hist
Frequency
(A)
v(sham_No-go) trace
v(sham_No-go) acorr
v(sham_No-go) hist
Frequency
(B)
v(tDCS_Go) trace
v(tDCS_Go) acorr
v(tDCS_Go) hist
a(1) trace
Frequency
(C)
v(sham_Go) trace
v(sham_Go) acorr
v(sham_Go) hist
Frequency
(D)
t(1) trace
t(1) acorr
t(1) hist
Frequency
(E)
t(2) trace
t(2) acorr
t(2) hist
Frequency
(F)
a(1) trace
a(1) acorr
a(1) hist
Frequency
(G)
a(2) trace
a(2) acorr
a(2) hist
Frequency
(H)
z(1) trace
z(1) acorr
z(1) hist
Frequency
(I)
z(2) trace
z(2) acorr
z(2) hist
Frequency
(J)

We calculated and summarized the trace, autocorrelation, and marginal posterior of each HDDM parameter to verify the convergence of the Markov-chain Monte Carlo chains and observed that: the traces for all HDDM parameters are fairly stationary, the autocorrelations are quite high as indicated by the long tails of the distributions, the approximation of the marginal posterior distribution is a normal distribution, the Geweke's statistic for each sub-figure was 'true', which meant there was no statistically significant difference between the start and end of the chain. Accordingly, the HDDM parameters that we calculated were properly converged.
